# Dip effect of asymmetric deformation characteristics for stope roof-pillar system

**DOI:** 10.1038/s41598-023-35006-w

**Published:** 2023-05-14

**Authors:** Tengda Huang, Binyu Luo, Yicheng Ye, Zhouhao Yuan, Pengcheng Li

**Affiliations:** 1 School of Resource and Environmental Engineering, Wuhan University of Science and Technology, Wuhan, 430081 Hubei China; 2 State Key Laboratory of Safety and Health for Metal Mines, Maanshan, 243000 Anhui China; 3 Hubei Key Laboratory for Efficient Utilization and Agglomeration of Metallurgic Mineral Resource, Wuhan, 430081 Hubei China; 4 School of Environmental Science and Engineering, Hubei Polytechnic University, Huangshi, 435003 Hubei China

**Keywords:** Solid Earth sciences, Mineralogy

## Abstract

In underground mining, the dip angle is one of the widely recognized factors that cause the asymmetric deformation of the goaf/stope roof, but characterizing the degree of asymmetric roof deformation is still a challenge. The goal of this research is to try to solve this problem with a theoretical model and numerical method. In an inclined ore seam, the mining load produces both normal and tangential effects on the inclined roof. A theoretical model was developed employing thin plate theory for enabling describe the asymmetric deformation of the roof caused by inclination. The proposed model describes not only the bending deformation state of the roof but also the deformation characteristics. Subsequently, the law of asymmetric deformation of roofs with varying inclinations was presented by numerical method. Under the same conditions, the numerical results of the asymmetric deformation of the roof are consistent with the theoretical results. Finally, the degree of asymmetrical deformation was characterized and quantified by the distance between the maximum subsidence point and the center of the roof. There exist three modes of asymmetric deformation, which are controlled by both dip angle and in-situ stress ratio. The results show that the shear load caused by dip angle is the root cause of asymmetric deformation of the roof. This study provides a theoretical basis for the asymmetric deformation control of the inclined roof.

## Introduction

Room and pillar mining is one of the essential methods for underground mining, which features a series of temporary or permanent pillars to support the overlying rock pressure and maintain the stability of the roof. In this mining method, workers conduct mining activities in a space (stope) supported by natural or artificial pillars. In the process, the space (stope) must be stable to ensure the safe mining of the ore body. After the ore body mining is completed, this space (goaf) also needs to be durable to control the surface collapse caused by the movement of the rock strata. Once the pillars in the goaf are destabilized, or the exposed area of the roof is too large, it may induce the deformation and instability of the roof and bring severe challenges to the safety of underground production. Therefore, studying the deformation characteristics of the stope/goaf roof is one of the significant ways to reveal its stability.

In flat seam mining, physical experiments, numerical simulations, and field monitoring are performed to reveal the deformation and failure modes. Fu et al.^1^ investigated the destabilization mechanism and stability control in stope mining roadway below the remaining coal pillars based on theoretical analysis, numerical simulation, and industrial testing. They found the law of stress transfer and proposed the corresponding support technology. The paraffin was melted to simulate the mining process in an actual three-dimensional physical experiment; the results show that the floor and roof deformation is symmetric, and the maximum flexure point is in the center of the excavation area^2^. The field monitoring and numerical method were used to explore deformation and failure patterns of gob-side entry driving heading adjacent and concluded theoretical criteria. Based on these results, a series of preventive techniques were developed to control the roof^3^. Abousleiman et al.^4^ investigated deformation mechanics and parametric sensitivities through the discrete element method. The results of parametric sensitivities confirmed that the self-supporting capacity is greatly influenced by entry depth and fine-scale bedding and then is determined by the stiffness and strength of intact materials. Zhu et al.^5^ carried out two physical similarity experiments to find the failure propagation of pillars and roofs. They divided it into two stages: the first stage is steady movement, and the second stage is the instability propagation stage. Roof collapse due to inefficient support of rock bolts is studied by field observations, numerical simulation, and lab tests. According to the situation, the improved support systems were designed to prevent such accidents^6,7^. In room and pillar mining, the remaining pillars will aggravate the damage to the roof. Thus, it is worthwhile to explore how the pillars affect the roof ^8,9^. Wang et al.^10,11^ drew stress circles of the entry roof in each stage of the whole life cycle based on the Mohr-Coulomb theory to judge whether the roof is damaged according to the strength envelope curve. The large deformation of the surrounding rock is not only affected by the stress but also by the movement of the upper roof. Sinha et al.^12,13^ proposed that three separate reinforcement factors are used to evaluate roof support plans. Mondal et al.^14^ established a predicted model that described the correlation between the cut-out distance of the continuous miner and the roof convergence through numerical simulations. This model was verified by observed data to determine the optimal cut-out distance. Chai^15^ and Pan et al.^16^ reveal the mechanism of ground pressure behavior induced by roof strata deformation using field testing technology and theoretical method.

In terms of theoretical research on roof deformation, Sun et al.^17^ evaluated the stability of roadways with a hard roof by beam theory and numerical method and compared it with field monitoring. Besides, the roof is modeled as an Euler-Bernoulli beam clamped by pillars and rocks to deduce the roof fracture process^18^. Zhang et al.^19^ explored failure and deformation characteristics in working face end by numerical simulation and thin plate theory. As the advance of the working face, the failure of the roof at the end of the goaf is periodic. According to the breaking characteristics, a thin plate model with three fixed sides and one free side is established. As the excavation of the ore body, the roof, regarded as a thin plate, is subjected to different boundary conditions. The stress distribution and fracture modes will change under different supports^20,21^. Ma et al.^22^ considered that pressure arch theory is used to derive the load borne by the goaf roof. Based on this load, the roof is modeled as a thin rectangular plate with four fixed support points to determine the optimum thickness. The thin plate theory is no longer applicable to this situation if the thickness is thick enough. These studies are aimed at the flat seam.

In general, the dip angle of geological strata will influence rock engineering characterizations, which is a recognized phenomenon in tunnel^23^ and mining projects^24^. Affected by the inclination of the orebody, the surrounding rock behavior of inclined orebody mining is different from that of horizontal orebody^25,26^, such as stress state, deformation law, and failure mode. Das et al.^27^ studied the influence of dip angle on the mechanical behavior of pillar and roof, the results show shearing effect and stress concentration is comparatively more severe at the dip side working. Li et al.^28^ used physical simulation and theoretical analysis to research roof structure stability formed by a three-hinged arch. With the dip angle increasing, the possibility of the roof structure becoming unstable due to large deformation decreases significantly, while the probability of the roof structure’s sliding instability above the tailgate increases. And a detailed field survey was proposed to evaluate the stability of inclined bedded surrounding rock mass and identify the high risking regions^29^. The influence of inclination on the deformation and failure of the roof is negligible^30–32^. Though existing research have revealed that the roof forms symmetrically in the strike direction and asymmetrical bending in the dip direction, the degree of dip influence on the asymmetric deformation of roofs needs to be further studied.

The goal of this research is to solve the issue mentioned above by combining theoretical and numerical methods. The theoretical model of asymmetric deformation of an inclined roof is developed by thin plate theory. Then the characteristics of roof deformation with variable dip angle are studied by numerical method. The numerical results and theoretical model results are compared and analyzed to look forward to the inclination effect of the roof asymmetric deformation mechanism. In order to further explore the law of asymmetric deformation, the coupling effects of in-situ stress ratio and dip angle on the magnitude and degree of roof deformation are studied. Finally, three deformation modes are proposed.

## The mechanical model of asymmetrical deformation of inclined roof

### Loads state of the roof and basic assumptions

**Figure 1:**
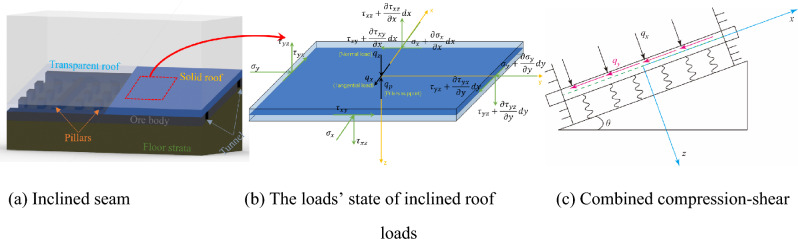
Inclined stope and stress state of stope roof.

In underground mining, the roof of the goaf/stope could be regarded as a thin plate, and then the thin plate theory is employed to investigate the deformation law. Existing research has proved that the stope/goaf roof mainly undergoes the expected load and bends symmetrically in flat seam mining. When the room and pillar method is used to extract the inclined ore seam, the goaf/stope roof is subjected to the load of the overburden rock mass, the horizontal lateral pressure, and the support of pillars, as shown in Fig. 1a. Affected by the inclination angle of the ore seam, the inclined roof presents normal load effect and load effect along the dip direction. The superposition of the two effects leads to asymmetric deformation of the roof. Thus, the stress state of the inclined roof could be decomposed along the normal and tangential directions, respectively, and decomposed into a combination of the normal load perpendicular to the roof and the tangential load parallel to the roof, as shown in Fig. 1b,c. The positive direction of the force is defined in this research. For normal stress, compression is used as the positive direction, and the positive direction is used in the same direction as the coordinate axis on the face, where the normal direction is positive for shear stress.

To simplify the model and calculations, some assumptions need to be made.

(1) The pillars will not yield under combined compression-shear loading and be regarded as an elastic foundation. (2) There is no large crack around the roof; the boundary condition is regarded as fixed support. (3) The tangential load only deforms the roof along the inclined direction and ignores the shear deformation.

### Calculation of the deflection caused by the normal load

**Figure 2:**
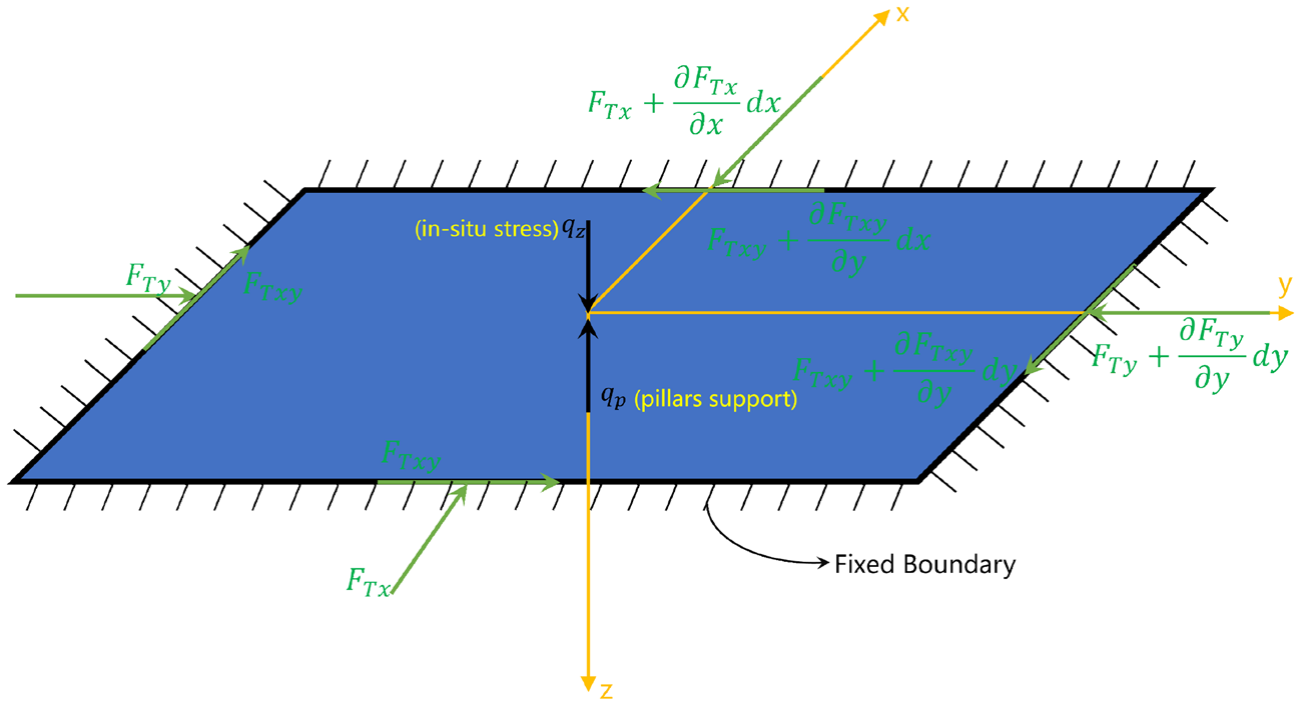
Mechanical model of the roof under normal load.

When the roof is subjected to normal load only, there is lateral ground pressure along the direction of roof thickness and the lower surface is subjected to the support force of the mine pillars. According to St. Venant’s theory^33^, the stress distribution on the four sides of the roof need not precisely satisfy the stress boundary conditions as long as the resultant force meets. Then,

1a $$ F_{Tx} = \int_{{ - \frac{h}{2}}}^{\frac{h}{2}} {\sigma_{x} d\delta } $$

1b $$ F_{Ty} = \int_{{ - \frac{h}{2}}}^{\frac{h}{2}} {\sigma_{y} d\delta } $$

1c $$ F_{Txy} = \int_{{ - \frac{h}{2}}}^{\frac{h}{2}} {\tau_{xy} d\delta } $$

where *σ*_*x*_ represents the normal stress in the *x* direction, *h* is the thickness of the roof, and *F*_*Tx*_ represents the resultant force of normal stress on the surface in the *x* direction. Similarly, *F*_*Ty*_ is the resultant force of normal stress on the surface in the *y* direction, and *F*_*Txy*_ are resultant force of shear stress around the boundary, as shown in Fig. 2. In addition, *q*_*n*_ is the support loads of the pillars, and its expression is

2 $$ q_{n} = \frac{{nA_{p} E_{p} }}{{A_{r} L}}w $$

where *n* is the number of pillars; *A*_*p*_ is the top surface area of a pillar; *A*_*r*_ is the area of the roof; *L* is the height of the pillar; *E*_*p*_ is the elastic modulus of pillar material, and *w* represents the deflection of the roof.

According to the thin plate theory, the equilibrium equation of the roof is given in the differential form,

3 $$ D\nabla^{4} w - \left( {F_{Tx} \frac{{\partial^{2} w}}{{\partial x^{2} }} + F_{Ty} \frac{{\partial^{2} w}}{{\partial y^{2} }} + 2F_{Txy} \frac{{\partial^{2} w}}{\partial x\partial y}} \right) = q_{z} - q_{n} $$

where *D* is bending stiffness, and it is obtained as

4 $$ D = \frac{{E_{r} h^{3} }}{{12\left( {1 - \mu^{2} } \right)}} $$

where *E*_*r*_ is the elastic modulus of the roof rock mass; *μ* is the Poisson’s ratio of the roof.

From the assumption (2), the boundary condition is considered as fixed support, and the boundary condition is expressed in terms of deflection as

5 $$ \left\{ \begin{gathered} w = 0 \hfill \\ \frac{\partial w}{{\partial x}} = 0 \hfill \\ \frac{\partial w}{{\partial y}} = 0 \hfill \\ \end{gathered} \right. $$

Let the roof deflection equation be expressed as follows,

6 $$ w = Cw_{m} $$

where *C* is constant, and *w*_*m*_ is an arbitrary function to ensure that the deflection satisfies the displacement boundary condition.

In addition, the Ritz method establishes the variational equation of the roof.

7 $$ \frac{{\partial V_{\varepsilon } }}{\partial C} = \iint_{{A_{r} }} {\left( {q_{z} - \frac{{2nA_{p} E}}{{A_{r} L}}w} \right)w_{m} dxdy} $$

where *Vε* is the deformation energy of the roof. Under the boundary condition of fixed support, the deformation energy could be given as,

8 $$ V_{\varepsilon } = \frac{D}{2}\iint_{{A_{r} }} {\left( {\nabla^{2} w} \right)^{2} dxdy} $$

Ulteriorly, the functional expression of the deflection is

9 $$ w = C\left( {x^{2} - a^{2} } \right)^{2} \left( {y^{2} - b^{2} } \right)^{2} $$

where *a* and *b* are half the length and width of the roof, respectively.

The expression of parameter *C* is obtained by substituting Eqs. (8) and (9) into Eq. (7),

10 $$ C = \frac{{256q_{z} }}{{450\left( {8Df + \frac{{nA_{p} E_{p} }}{{A_{r} L}}g} \right)}} $$

where

11 $$ \left\{ \begin{gathered} f = \frac{2048}{{11025}}\left( {7a^{4} + 4a^{2} b^{2} + 7b^{4} } \right) \hfill \\ g = \frac{65536}{{99225}}a^{4} b^{4} \hfill \\ \end{gathered} \right. $$

Then, substituting Eqs. (10) and (11) into Eq. (9), the deflection expression of the roof is gained under normal load,

12 $$ w = \frac{{256q_{z} }}{{450\left( {8Df + \frac{{nA_{p} E_{p} }}{{A_{r} L}}g} \right)}}\left( {x^{2} - a^{2} } \right)^{2} \left( {y^{2} - b^{2} } \right)^{2} $$

### Calculation of the displacement caused by tangential load

According to assumption (3), on the boundary opposite to the direction of tangential stress (i.e., the normal of one end boundary along the inclined direction), the ground stress will prevent the roof from moving, on the other boundary, as the new displacement space caused by the roof deformation, the rear rock layer does not affect the longitude deformation under the ground stress. For the transverse flexural deformation of the roof, considering the clamping effect of the upper and lower strata, the roof rock has no deformational space, thus, is fixed around. The longitudinal deformation is the deformation of the roof strata itself, which needs further solving. Since the longitudinal deformation is minimal, the influence of the rock mass at the boundary of the strike is ignored. Therefore, the equilibrium equation of the roof is as follows,

13 $$ \frac{{\partial F_{x} }}{\partial x}dx = q_{x} dx $$

The *F*_*x*_ obtained from the boundary condition is

14 $$ F_{x} = q_{x} x + \frac{{q_{x} }}{2} $$

From physical and geometric equations in elasticity, the expressions are given as,

15 $$ \varepsilon_{x} = \frac{{\partial x_{0} }}{\partial x} = \frac{{F_{x} }}{Eh} $$

16 $$ x_{0} = \frac{1}{Eh}\int {F_{x} dx} $$

By combining Eqs. (14) and (16), the displacement of the roof along the inclination direction is obtained under the tangential load as follows,

17 $$ x_{0} = \frac{{q_{x} }}{2Eh}x^{2} + \frac{{q_{x} b}}{Eh}x - \frac{{3q_{x} b^{2} }}{3Eh} $$

### Calculation of the displacement caused by normal and tangential load

Equation (17) indicates that the displacement occurs along the inclination direction of the roof, which is equivalent to each point on the roof offset along this direction. Compared with the displacement under the normal load (Eq. (12)), when the roof is subjected to combined normal load, and tangential load, the coordinate of the roof becomes (*x* + *x*_0_) in the inclined direction. At this time, the deflection equation of the roof under combined compression-shear load is written as

18 $$ w = C\left( {\left( {x + x_{0} } \right)^{2} - a^{2} } \right)^{2} \left( {y^{2} - b^{2} } \right)^{2} $$

where *C* is determined by Eq. (10), and the distance *x*_0_ is determined by Eq. (17). From Eq. (18), it can be known that the maximum displacement position is at the coordinate (− *x*_0_, 0).

Further, the expression of vertical displacement is obtained from Eq. (18),

19 $$ w_{v} = C\left( {\left( {x + x_{0} } \right)^{2} - a^{2} } \right)^{2} \left( {y^{2} - b^{2} } \right)^{2} \cos \theta $$

where *θ* is the dip angle of the seam.

## The numerical simulation of asymmetrical deformation of the roof

### Engineering background

**Figure 3:**
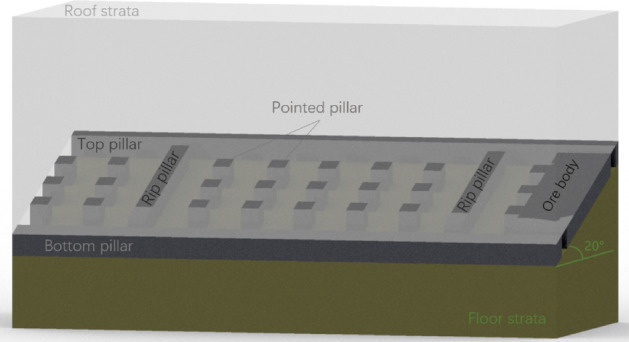
Layout plan of mine room and pillar.

A gently inclined phosphate deposit is regarded as the research object to study the dip effect on the deformation behavior of the roof by numerical method. The phosphate ore layer is located at the Doushantuo Formation of the Upper Aurignacian. The orebody is stratified and produced in line with the occurrence of the strata, without horse-stone. The occurrence elevation of the ore layer is from 480 to 1130 m; the buried depth is about 250–600 m, and the dip of the seam varies from 0° to 25°. And the thickness of the seam is equal to from 0.55 to 9.13 m. The roof is dolomite containing phosphorus bands and phosphorus nodules, and the rock layer is medium-thick and stable in distribution. Some areas of the floor are mudstone and shale containing banded phosphorite or phosphorus masses, and the rest are phosphate-bearing powdery dolomite. According to the occurrence characteristics of the ore layer, the room and pillar method was selected. A rip pillar of 5 m in width is left between the ore blocks, the top and bottom pillars are arranged along the direction, the top pillar is equal to 5 m, and the bottom pillar is 5 m. The sectional transportation roadway is arranged in the top and bottom pillars. The room is arranged horizontally along the strike in the block, and regular point pillars are left in the goaf. The length and width of the point pillar are 5 m × 5 m, the interval along the strike is equal to 10 m, and the interval along the dip is 8 m, as shown in Fig. 3.

### Estimation of the mechanical parameters of rock mass

The Hoek-Brown criterion^34,35^ is used to estimate the strength parameters of rock mass and is given as,

20 $$ \sigma_{1} = \sigma_{3} + \sqrt {m\sigma_{c} \sigma_{3} + s\sigma_{c}^{2} } $$

In the above formula, the uniaxial compressive strength *σ*_*mc*_ and tensile strength *σ*_*mt*_ of rock mass are obtained by making *σ*_3_ = 0 and *σ*_1_ = 0, respectively.

21 $$ \left\{ \begin{gathered} \sigma_{mc} = \sqrt s \sigma_{c} \hfill \\ \sigma_{mt} = \frac{1}{2}\sigma_{c} \left( {m - \sqrt {m^{2} + 4s} } \right) \hfill \\ \end{gathered} \right. $$

And the relationship between the constant *m*, *s*, and *RMR* is given as,

22 $$ \left\{ \begin{gathered} \frac{m}{{m_{i} }} = \exp \left( {\frac{RMR - 100}{{14}}} \right) \hfill \\ s = \exp \left( {\frac{RMR - 100}{6}} \right) \hfill \\ \end{gathered} \right. $$

where *m*_*i*_ is a constant for intact rock, the strength of rock mass can be evaluated by Eqs. (20) ~ (22).

In addition, the elastic modulus of rock mass is calculated by the empirical equation proposed by Hoek and Diederichs^36^.

23 $$ E_{m} = E_{i} \left( {0.02 + \frac{1 - D/2}{{1 + \exp \left( {(60 + 15D - (RMR - 5))/11} \right)}}} \right) $$

where *E*_m_ represents the elastic modulus of rock mass, *E*_*i*_ represents the elastic modulus of intact rock; and *D* is the blasting influence coefficient (0 ~ 1).

According to the mine geological report and laboratory test results, Eqs. (20) ~ (23) assess the mechanical parameters of rock mass, which derives the parameters of each layer, as shown in Table 1.

**Table 1:** Mechanical parameters of rock mass.

Lithology	Elastic modulus/GPa	Poisson’s ratio	Cohesive/MPa	Friction/°	Tensile strength/MPa
Dolomitic limestone	5.20	0.30	2.1	28.1	1.5
Argillaceous limestone	0.90	0.27	0.8	26.0	1.1
Silty dolomite	1.80	0.33	2.2	35.8	1.8
Argillaceous dolomite	2.90	0.22	1.8	27.4	0.7
Dolomite	0.60	0.22	3.0	35.1	2.3
Phosphate rock	0.65	0.27	5.0	34.2	2.8
Silty dolomite	6.91	0.25	2.2	36.1	1.8
Potash shale	1.30	0.23	1.6	41.5	0.9

### Establishment of deposit model

**Figure 4:**
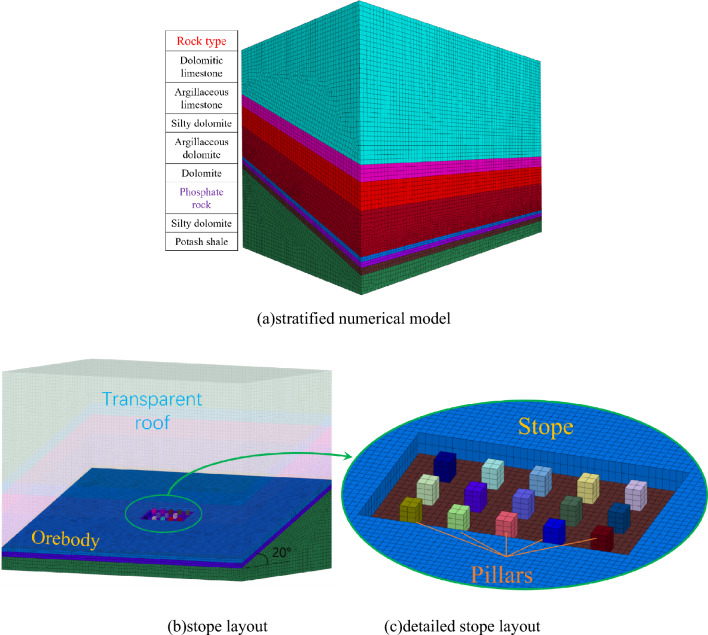
Deposit numerical model.

To explore the dip effect on the displacement of the goaf roof, the FLAC^3D^ software (Fast Lagrangian Analysis of Continua in 3-Dimensions)^37^ is used to establish numerical deposit models with dip angles of 0°, 10°, 20°, 30°, and 40°, respectively. To the dip of the seam is the negative *x*-axis. The strike length of the stope is 85 m, and the width along the inclined direction is 47 m. There are 15 pillars left in the goaf, whose length and width are 5 m × 5 m, and the height of the pillar is 6 m. The deposit model is divided into 8 layers from bottom to top, and the model dimensions are 425 m × 282 m × 208 m, as shown in Fig. 4.

The average overlying strata thickness of the seam is 300 m. The overlying rock load is about 6.7 MPa, and the ratio of in-situ stress is equal to 1.2, then the horizontal ground stress applied is 8.2 MPa. The fixed support at the bottom and around the model prevents the model from moving. The model is assigned to the Mohr-Coulomb model.

### The numerical results of asymmetrical deformation for the roof

**Figure 5:**
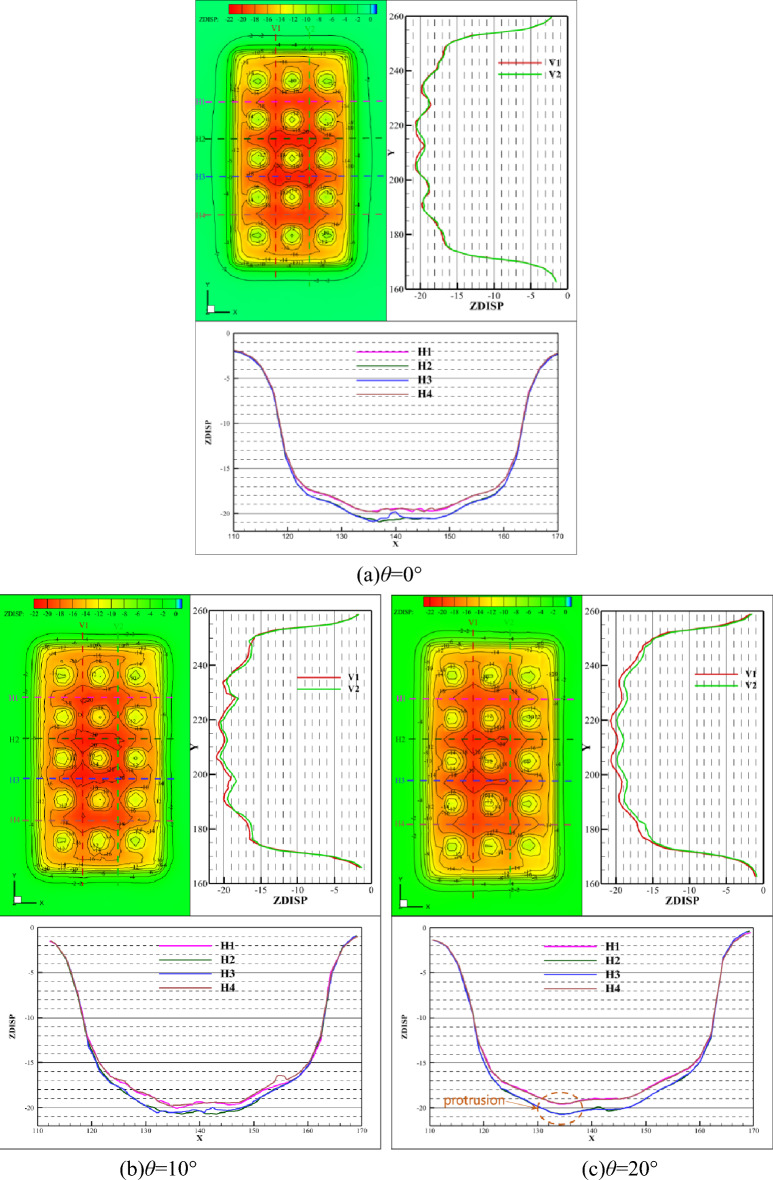

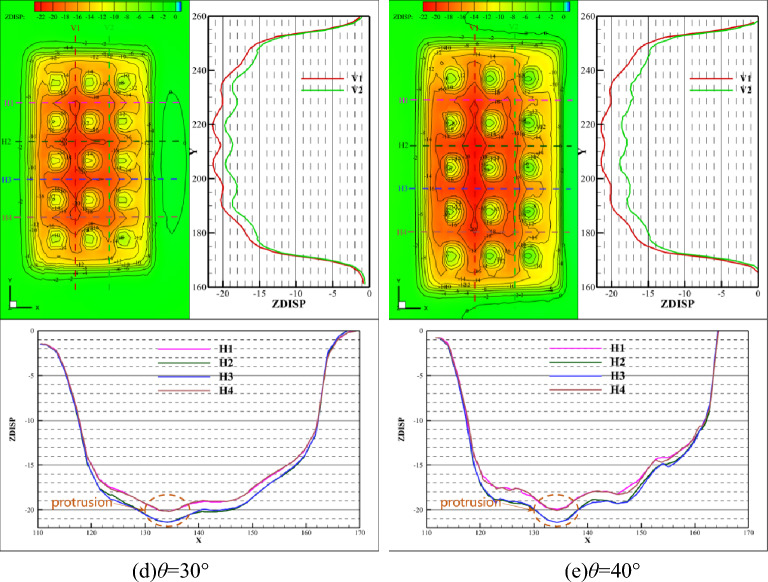
Vertical displacement of the roof under different dip angles.

Figure 5 shows the vertical displacement contour on the lower surface of the roof under the dip angles of 0°, 10°, 20°, 30°, and 40°, respectively. Two sampling lines, V1 and V2, are extracted equidistantly on both sides of the centerline of the roof to acquire the law of deformation in the direction of the strike. In the same way, in the direction of inclination, four lines, H1, H2, H3, and H4, are extracted to obtain the displacement characteristics. All the lines are on the lower surface of the roof, and there are 200 points on each line. The inclination direction of the roof is along the negative *x*-axis. The curves on the right side of the contour are vertical displacements on the two lines (V1 and V2) along the strike direction. The curves below the contour are vertical displacements on the four lines (H1, H2, H3, and H4) along the inclination direction. When the dip angle is equal to 0°, the vertical displacement of the roof is more than 18 cm. The negative sign indicates the displacement direction. The vertical displacement in the center of the roof is the largest and reaches 21 cm. Taking the central axis of the roof as the center, the displacement in the strike direction and inclination direction are equal, respectively, and the distribution law is consistent, as shown in Fig. 5a. This means that the deformation of the roof is symmetrical when the dip angle is equal to 0°. As the dip angle increases, the vertical displacement distributes symmetrically as before, but the maximum displacement gradually moves downwards along the inclination direction. With the center of the roof as the boundary, the displacement on the tilted-up side is smaller than the displacement on the titled-down side (the displacement on the V1 sampling line is greater than the V2 sampling line). In the inclination direction, vertical deformation is no longer symmetrical along the centerline. With the increase of dip angle, the maximum vertical displacement decreases gradually and offsets downward to the inclined direction. Furthermore, the vertical displacement contour where pillars support forms the shape of a “tree ring.” The left side of the “annual ring” is gradually dense, and the displacement gradient becomes larger, which indicates that the pillar is also asymmetrically deformed.

**Figure 6:**
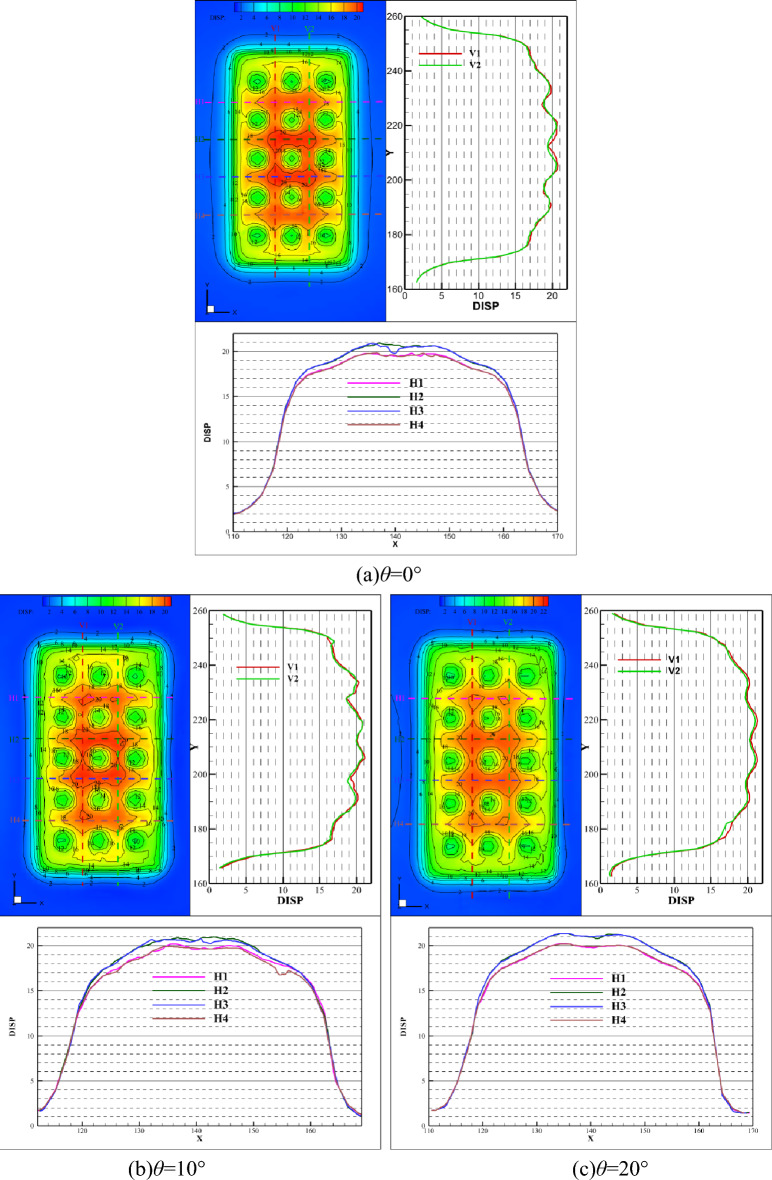

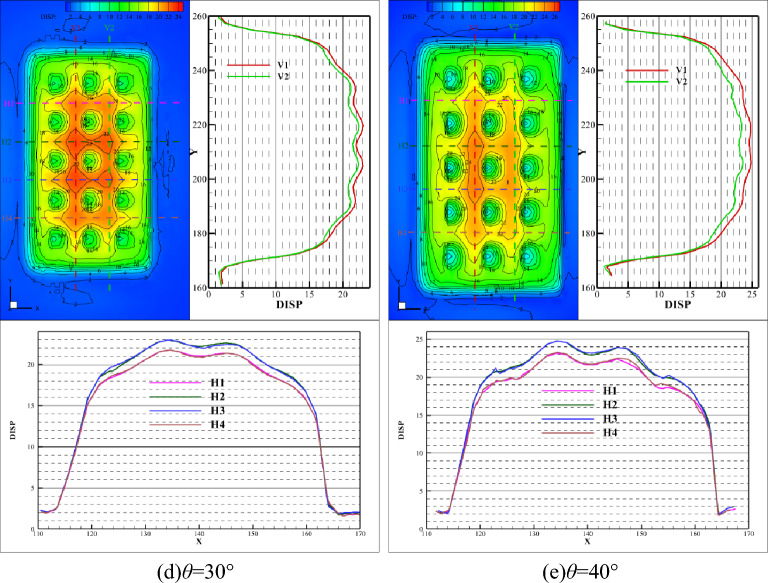
Total displacement of the roof under different inclinations.

Figure 6 shows the total displacement distribution on the lower surface of the goaf roof with a dip angle of 0°, 10°, 20°, 30°, and 40°, respectively. When the dip angle *θ* is equal to 0°, the displacement mainly occurs in the vertical direction, and the total displacement is similar to the vertical displacement. As the increase of dip angle, the total displacement increases appreciably, while the vertical displacement changes little relatively. The deformation law is in accordance with that in the vertical direction, and the total displacement continues to reflect symmetry along the strike direction and asymmetry along the inclination direction.

## Discussion and analysis

### Analysis of deformation characteristics of the inclined roof along strike and dip

**Figure 7:**
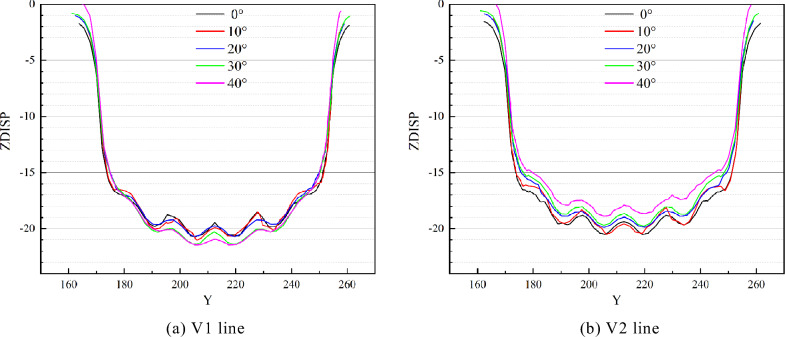
Comparison of vertical displacement in strike direction.

**Figure 8:**
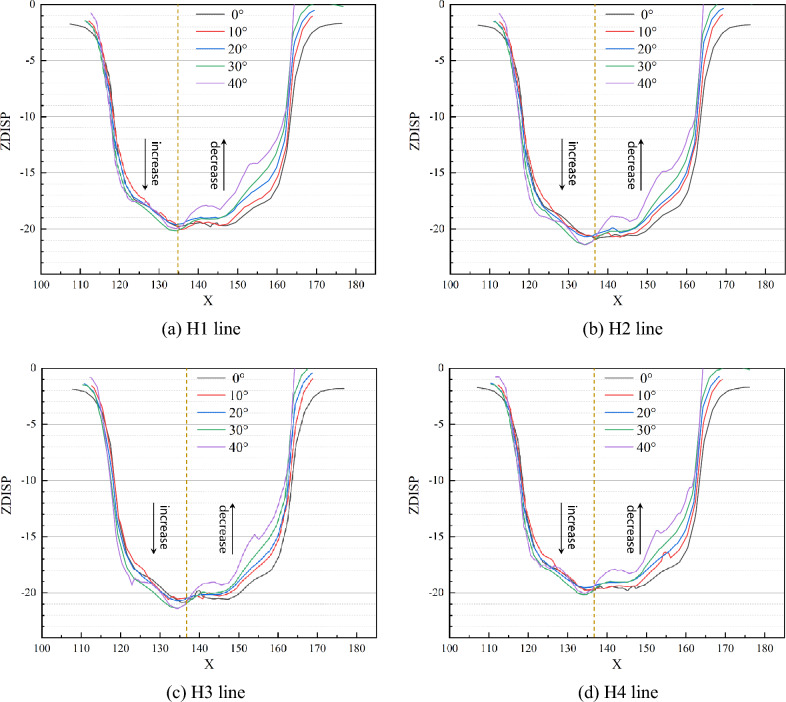
Comparison of vertical displacement in the inclination direction.

Figures 7 and 8 illustrate the comparison curve of vertical displacement on two lines in the strike direction (V1 and V2) and four lines in the inclination direction (H1, H2, H3, and H4) under different dip angles, respectively. On the V1 of line, the vertical displacement increases with the increase of inclination angle, while the law is the opposite on the V2 line. And the displacement becomes smaller, as shown in Fig. 7. Bounded by *x* = 136 m, the displacement of the left side increases with the increase of the dip angle, while the displacement of the right decreases with the increase of the dip angle, as shown in Fig. 8. It indicates that dip angle offsets the vertical displacement and induces aggravated uneven deformation, which means that the asymmetrical deformation of the roof is intensified.

**Figure 9:**
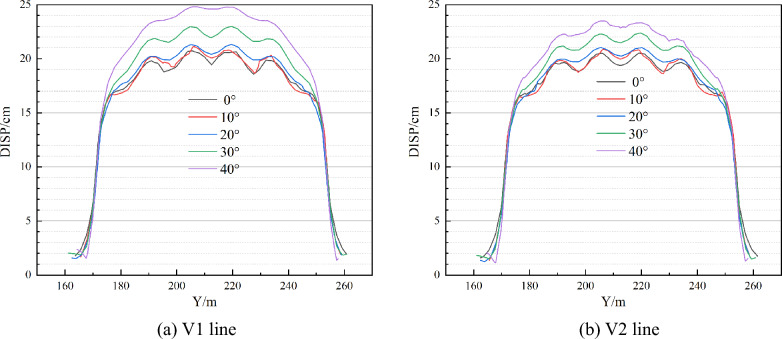
Comparison of total displacement in strike direction.

**Figure 10:**
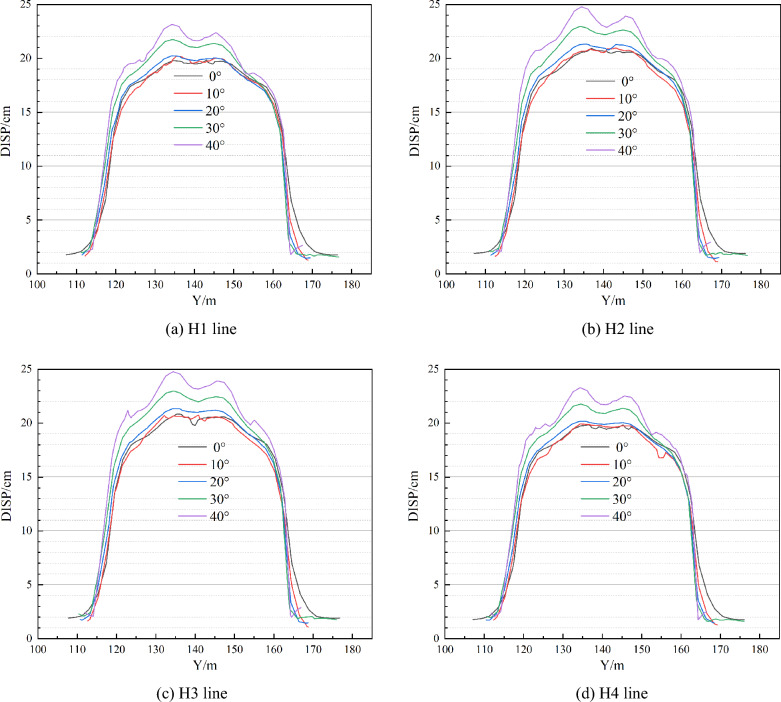
Comparison of total displacement in the inclination direction.

Figures 9 and 10 illustrate the comparison curve of total displacement on two lines in the strike direction (V1 and V2) and four lines in the inclination direction (H1, H2, H3, and H4) under different dip angles. The total displacement increases with the increase of dip angle both in strike and inclination. The reason for this phenomenon is that the combined action of horizontal and vertical in-situ stress on the upper surface of the roof causes displacement. And the larger the horizontal in-situ stress, the greater the combined force on the roof and the greater the total displacement. If the dip angle increases to a certain degree, at this time, the increase of horizontal in-situ stress cannot compensate for the decrease of vertical in-situ stress, which would lead to a decrease in total displacement.

### Theoretical analysis of asymmetric deformation characteristics of inclined roof

According to the decomposition of force, the normal load acting on the roof is obtained,

24 $$ q_{z} = \frac{{\sigma_{v} \cdot 4ab \cdot \cos \theta \cdot \cos \theta }}{4ab} + \frac{{\sigma_{h} \cdot 4ab \cdot \sin \theta \cdot \sin \theta }}{4ab} = \sigma_{v} \cos^{2} \theta + \sigma_{h} \sin^{2} \theta $$

In the same way, the tangential load acting on the roof is obtained:

25 $$ q_{x} = \frac{{\sigma_{v} \cdot 4ab \cdot \cos \theta \cdot \sin \theta }}{4ab} + \frac{{\sigma_{h} \cdot 4ab \cdot \sin \theta \cdot \cos \theta }}{4ab} = \frac{1}{2}\left( {\sigma_{v} + \sigma_{h} } \right)\sin 2\theta $$

where *σ*_*v*_ represents the vertical in-situ stress before mining; *σ*_*h*_ represents the horizontal in-situ stress before mining.

Taking the same parameters as numerical simulations in Table 1, the theoretical model is proposed to calculate the displacement of the inclined roof. The parameter values with *a* = 23.5 m, *b* = 42.5 m, Eq. (24), and Eq. (25) are substituted into Eqs. (12) and (17), respectively. The deformation equation of the roof is obtained,

26 $$ w = C\left( {\left( {Ax^{2} + Bx + P} \right)^{2} - 23.5^{2} } \right)^{2} \left( {y^{2} - 42.5^{2} } \right)^{2} $$

where the parameter *C* determines the magnitude of vertical displacement; parameter *A* determines the density of the contour lines at equal intervals; parameter *B* determines the relationship between the length of the major and minor axis of the contour ellipse; The magnitude of the parameter *P* determines the offset distance of the maximum displacement point; The sign symbol determines the direction of migration. The magnitude of parameter *C* depends on the normal load acting on the roof. The magnitude of parameters *A*, *B,* and *P* depends on the longitudinal displacement related to the tangential load of the roof. From Eqs. (24) and (25), the magnitude of the normal load and tangential load acting on the roof are determined by the inclination of seam, horizontal, and vertical in-situ stress. These factors can be expressed by the dip angle and the ratio of in-situ stress.

**Figure 11:**
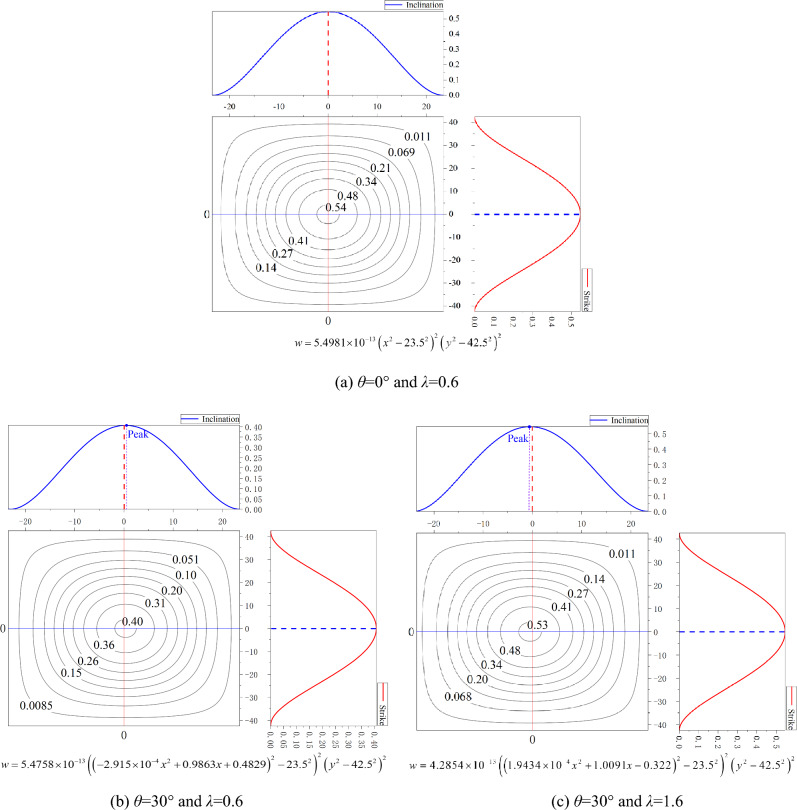
Contour map of roof deflection.

The contour map of the roof can be drawn from Eq. (26), as shown in Fig. 11. Figure 11a–c shows the roof bending under the ratio of in-situ stress is equal to 0.6 or 1.6, and the dip angle is 0° or 30°, respectively. When the seam is flat, the maximum deformation point is at the center of the roof without migration, as shown in Fig. 11a. The maximum displacement point moves in the direction of the positive *x*-axis, namely the inclination upward direction, as shown in Fig. 11b. However, the point moves downward in Fig. 11c (the curve above the contour map). The maximum deformation point has no excursion in the strike direction (the curve is on the right of the contour map). From the point of view of subsidence, the displacement is largest when the dip angle is equal to 0°, followed by the case of Fig. 11c, and the smallest displacement is shown in Fig. 11b.

**Figure 12:**
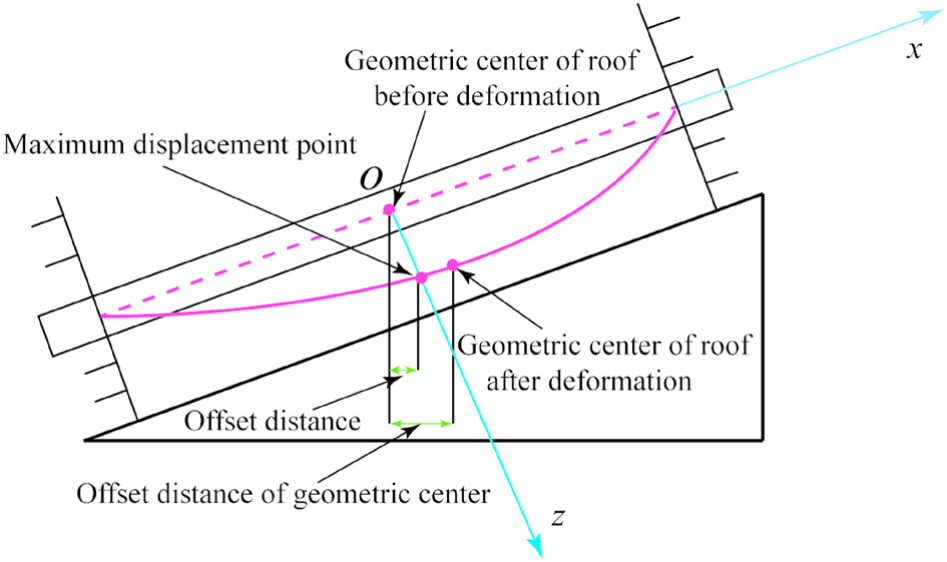
Schematic diagram of the offset distance of the roof.

To analyze the asymmetry of the roof deformation, the distance between the maximum subsidence point of the roof and the center of the roof is used to characterize the degree of asymmetrical deformation. This distance is called “the offset distance,” as shown in Fig. 12, and is defined as,

27 $$ x_{off} = \frac{1}{2}\Delta x_{max} - \Delta x_{0} $$

where Δ*x*_*off*_ is the offset distance; Δ*x*_*max*_ is the maximum longitudinal compression of the roof boundary, i.e., the boundary end with free deformation space; Δ*x*_0_ is the distance between the geometrical center before and after deformation, i.e., the offset distance of the geometrical center. If the offset distance is larger, the asymmetrical deformation is more serious; on the contrary, the asymmetrical deformation is smaller.

**Figure 13:**
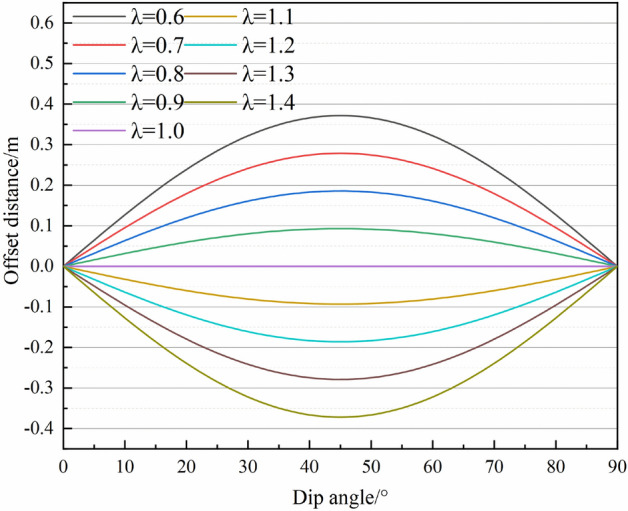
The offset distance of the maximum subsidence point.

According to the definition of the offset distance, the relationship between offset distance and dip angle and in-situ stress ratio is obtained as shown in Fig. 13. As can be seen from the diagram, with the ratio of in-situ stress λ = 1.0 as the boundary, the maximum subsidence point offset along both sides of the inclination direction respectively. When the ratio of in-situ stress λ is equal to 1.0, The maximum subsidence point has no excursion, namely symmetrical deformation. When λ > 1.0, the maximum subsidence point moves downward along the inclination direction, i.e., the greater the ratio of in-situ stress is, the farther the offset distance is, and the more serious the degree of asymmetrical deformation. When λ < 1.0, the maximum subsidence point moves upward along the inclination direction, i.e., the smaller the ratio of in-situ stress is, the more serious the degree of asymmetrical deformation is. From the point of view of the dip angle, with *θ* = 45° as the turning point, when *θ* is smaller than 45°, the offset distance increases as the dip angle increases. Conversely, when *θ* is greater than 45°, the offset distance moves backward as the dip angle increases. It can be concluded from Eq. (25) that the sine function reaches its maximum at 90°. When the dip angle *θ* increases by two times, the change of tangential load turns at 45°, which results in a turning point in the offset distance of the maximum subsidence point.

**Figure 14:**
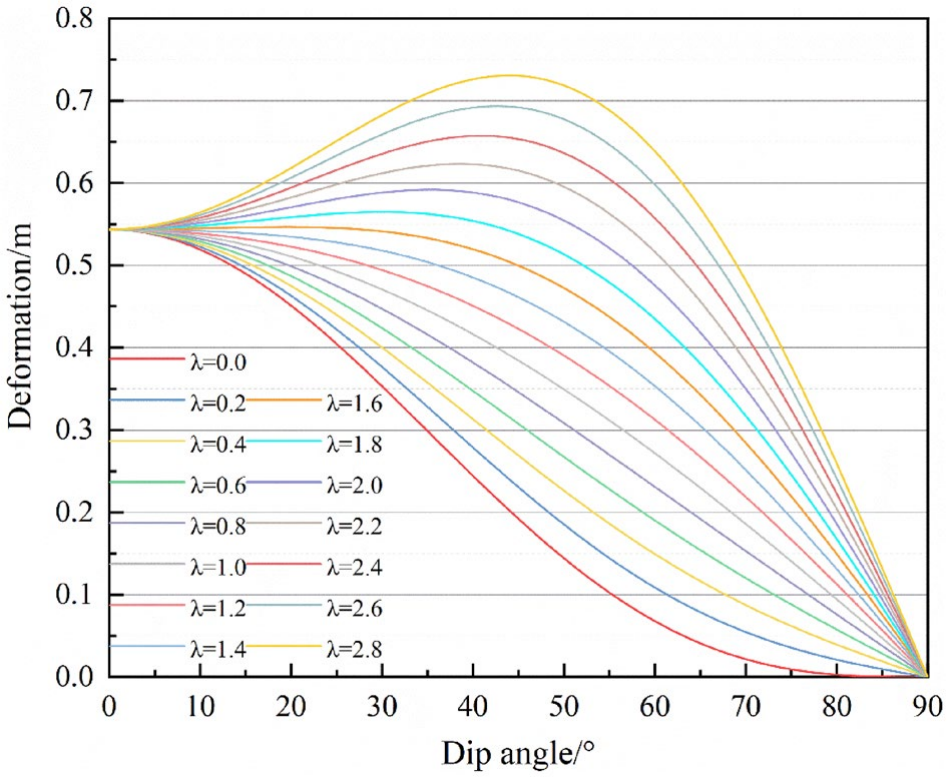
Roof deformation characteristics.

Figure 14 presents the relationship between the vertical displacement and the ratio of in-situ stress. When the ratio of in-situ stress *λ* is less than 1.6, the vertical displacement decreases with the increase of dip angle. While *λ* is greater than 1.6, this displacement increases first and then falls, and finally, when the dip reaches 90°, the roof has no vertical displacement. When the ratio of in-situ stress is greater than 1.0, normal load increase with the increase of dip angle. If *λ* is less than 1.0, the normal load continuously decreases. The normal load directly depends on the deflection, while the transformation of deflections to vertical displacement needs to be multiplied by the cosine function of the dip angle. Therefore, the vertical displacement is not synchronized with the change of the normal load. The cosine function decreases monotonously in the range of 0° ~ 90°, which leads to the maximum deformation in the vertical direction predominantly reducing. It increases only when the ratio of in-situ stress is relatively large. In the direction of roof inclination, when *λ* > 1.0, the maximum displacement of asymmetrical deformation of the roof occurs in the position of inclined downward. When *λ* < 1.0, the maximum displacement occurs in the place of inclined upward. The degree of asymmetrical deformation increases first and then decreases as the increase of dip angle, and reaches the peak at 45°when *λ* is a constant.

### Symmetric deformation characteristics of the inclined roof under the influence of in-situ stress ratio and dip angle

An in-depth exploration of the displacement variation with the in-situ stress ratio and inclination is the content of understanding the asymmetric deformation of the roof. Figure 14 illustrates that the vertical displacement has an increased stage. When the ratio of in-situ stress is comparatively large, the angle at which the vertical displacement reaches its peak also changes with the ratio of in-situ stress. To find these laws, Eq. (24) is derived for the inclination angle *θ*.

28 $$ q_{z}^{\prime } \left( \theta \right) = \left( {1 - \lambda } \right)\sigma_{v} \sin 2\theta $$

From Eq. (28) that the derivative is positive at *θ* ∈ [0°, 90°] when the ratio of in-situ stress is larger than 1.0. Therefore, the normal load and total displacement increase with the increase of the dip angle. If the ratio of in-situ stress is smaller than 1.0, the total displacement continuously decreases.

To conclude the variation law of vertical displacement with inclination angle, the ratio of in-situ stress is regarded as a constant, then the differential of (*q*_*z*_ cos*θ*) is obtained,

29 $$ \left( {q_{z} \cos \theta } \right)^{\prime } = \sigma_{v} \left( {2\lambda \cos^{2} \theta \sin \theta - 3\cos^{2} \theta \sin \theta - \lambda \sin^{3} \theta } \right) $$

The in-situ stress *σ*_*v*_ is constant and has no influence on the variation law of the derivative. Let

30a $$ f\left( \theta \right) = 2\lambda \cos^{2} \theta \sin \theta - 3\cos^{2} \theta \sin \theta - \lambda \sin^{3} \theta $$

30b $$ f^{\prime}\left( \theta \right) = \left( {2\lambda - 3} \right)\cos^{2} \theta \sin \theta - \lambda \sin^{3} \theta $$

Due to the inclination angle *θ* ∈ [0°, 90°], the slope at the two ends,

31 $$ \left\{ \begin{gathered} f^{\prime}\left( 0 \right) = 0 \hfill \\ f^{\prime}\left( {\frac{\pi }{2}} \right) = - \lambda \hfill \\ \end{gathered} \right. $$

Obviously, when *θ* = 0°, the vertical displacement slope equals 0. And when *θ* = 90°, the slope is associated with the ratio of in-situ stress. This law is consistent with the variation as shown in Fig. 12.

Let the derivative in Eq. (30b) be equal to zero, and

32 $$ \lambda = \frac{{3\cos^{2} \theta }}{{2\cos^{2} \theta - \sin^{2} \theta }} $$

**Figure 15:**
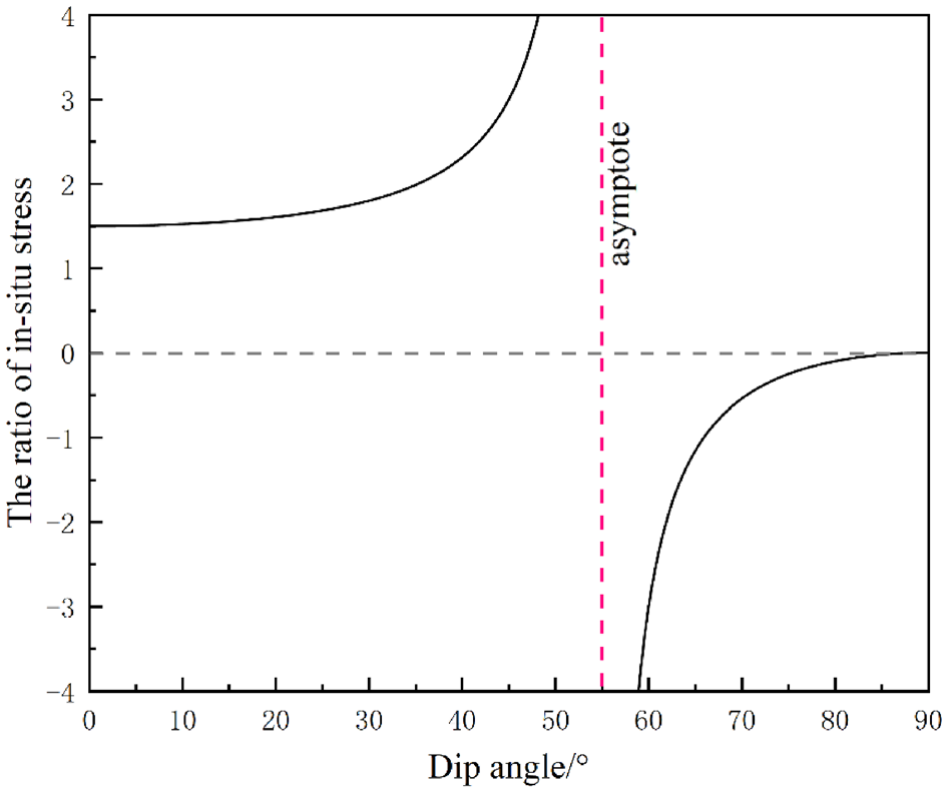
The ratio of in-situ stress corresponding to the maximum vertical displacement under each dip angle.

The function curve of Eq. (32) was presented as shown in Fig. 15. The curve is divided into two parts with an inclination angle of 55° as the asymptote. The minimum value of *λ* is equal to 1.5 at the left branch curve, and the maximum is equal to 0 at the right branch curve. The results show that Eq. (30b) has two zero values when the ratio of in-situ stress is greater than 1.5. The first zero corresponds to a dip angle of 0°. The second zero is the inclination of the ratio of in-situ stress corresponding to the intersection of the left branch in Fig. 15, which can be obtained from Eq. (32). The case of two zeros contains two meanings, one is that the vertical displacement has a rising phase. The other is that the zero point is the abscissa corresponding to the vertex of the rising stage of the curve, i.e., the inclination angle corresponding to the maximum vertical displacement of the roof. The function has two zeros, indicating that its monotonicity increases and decreases, that is, when the ratio of in-situ stress is greater than 1.5, for any *λ*, the maximum vertical displacement increases or decreases with the addition of inclination angle. When the ratio of in-situ stress is between 0 and 1.5, the maximum vertical displacement of the roof decreases with the increase of inclination angle.

**Figure 16:**
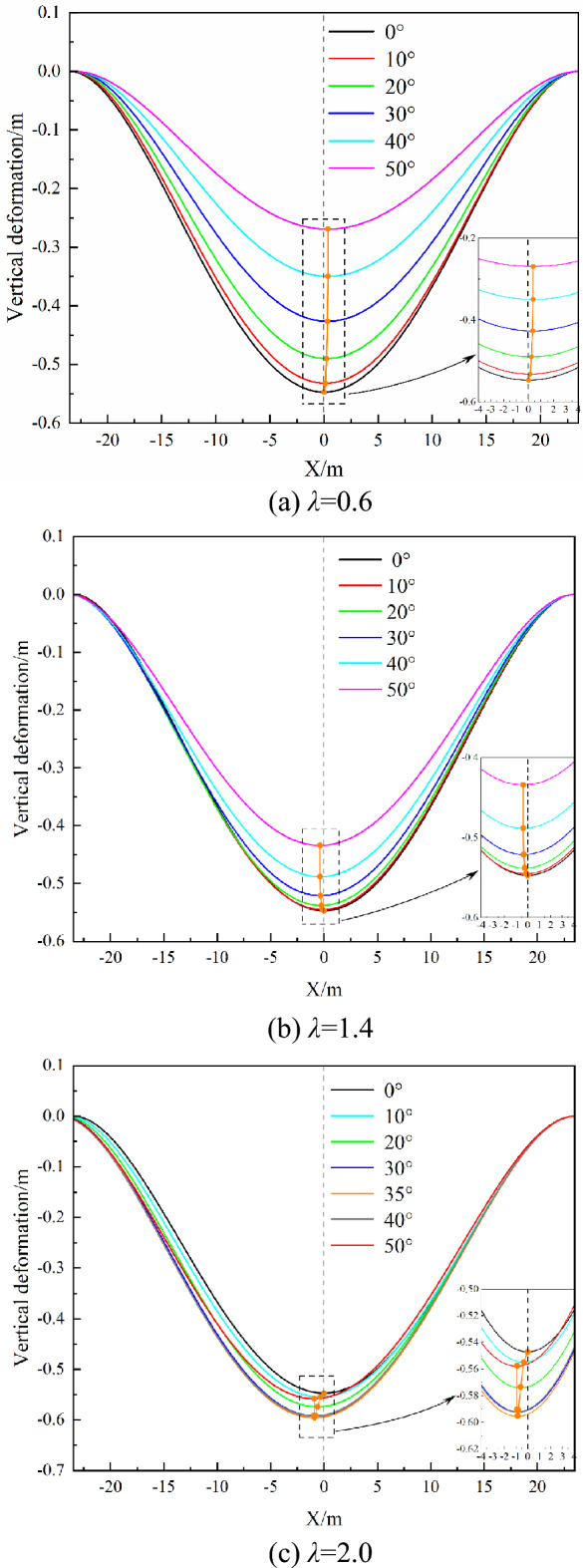
Three modes of asymmetric deformation of the roof.

As the aforementioned show that the roof asymmetrical deformation mode will be determined by the ratio of in-situ stress and inclination angle, and the mode of asymmetric deformation of the roof will be presented. In terms of the maximum vertical displacement deviating from the central position of the roof (As shown in Fig. 13), the demarcation point is that the ratio of in-situ stress *λ* is equal to 1.0. when *λ* is greater than 1.0, the maximum vertical displacement shifts downward and vice versa. As far as the variation of the maximum vertical displacement is concerned (As shown in Fig. 14), the ratio of in-situ stress *λ* is equal to 1.5 is the demarcation point of the maximum vertical displacement. When *λ* is greater than 1.5, the maximum vertical displacement increases and decreases with the change in the inclination angle. When *λ* is less than 1.5, the maximum vertical displacement decreases with the increase of the inclination angle. Thus, to directly present the pattern of asymmetric deformation of the roof in the above law, when the ratio of in-situ stress *λ* is equal to 0.6, 1.4, and 2.0, respectively, the function graph was drawn, i.e., the vertical displacement along the centerline of the roof, as shown in Fig. 16. When the ratio of in-situ stress is less than 1.0, the maximum vertical displacement point moves in the positive direction of the *x*-axis, namely the roof tilt upward. When the ratio of in-situ stress is greater than 1.0, the maximum vertical displacement point moves in the negative direction of the *x*-axis, namely the tilt down, as shown in Fig. 16b,c. When the ratio of in-situ stress is greater than 1.5, the maximum vertical displacement point first moves downward with the increase of the dip angle, and upward when the dip angle is greater than 35° (Fig. 16c). When the ratio of in-situ stress is less than 1.5, under any the ratio of in-situ stress, the maximum vertical displacement decreases with the increase of inclination angle.

## Conclusions

In this research, the dip effect mechanism of asymmetric deformation of the goaf roof was investigated by theoretical and numerical methods. A theoretical model was proposed for describing the asymmetric deformation of the roof. Then the degree of asymmetric deformation was characterized, and several modes of asymmetric deformation were revealed. The main conclusions have been summarized as followings.

1. A theoretical model was developed to describe the asymmetric deformation of the roof caused by inclination. The proposed model incorporates the deformation effect of the roof under the combined action of normal compression load and tangential shear load. Normal compression load bends the roof, while the tangential shear load causes the roof deformation along the inclined direction and changes the symmetry of the roof deformation. These two kinds of deformation effects are superimposed together, resulting in asymmetric roof deformation.
2. The numerical method was used to investigate the asymmetric deformation characteristics of the roof with varying dip angles. The vertical displacement changes less, and total displacement increases faster as the increase of dip angle. The influence of horizontal displacement is small in the goaf with a small inclination, but the closure of the goaf with a large inclination arises from it. In terms of displacement distributions, inclination leads to the displacement of the roof being symmetrical along the strike and asymmetrical along the inclined direction. The numerical results were compared with the theoretical model, which shows that the roof deformation laws obtained by the two methods are consistent.
3. The distance between the maximum subsidence point and the center of the roof was defined to characterize the degree of asymmetric deformation, the larger the distance is, the greater the degree of asymmetric deformation is; on the contrary, the smaller the asymmetric deformation. When λ > 1.0, the maximum deformation position shifts downward along the roof inclination, and the greater the degree of asymmetric deformation is with the increase of the ratio of in-situ stress. Similarly, when λ < 1.0, the maximum deformation position shifts upward to the tilt, and the smaller the initial stress ratio, the greater the degree of asymmetric deformation. When λ is constant, the degree of asymmetric deformation increases at first and then decreases with the increase of inclination angle. The degree of asymmetric deformation is the most when the dip angle is equal to 45°.
4. There exist three modes of asymmetric deformation, which are determined by both dip angle and in-situ stress ratio λ. When λ < 1.0, the maximum subsidence position shifts upward along the dip, and the maximum vertical displacement decreases with the increase of the dip angle. When 1.0 < λ < 1.5, the maximum subsidence position migrates downward along the dip, and the maximum vertical displacement decreases with the inclination increase. When λ > 1.5, the maximum subsidence position is shifted downward along the dip. With the rise of the dip angle, the maximum vertical displacement gradually increases and then decreases.

## Data Availability

The datasets used and analyzed during the current study are available from the corresponding author on reasonable request.
